# Living Polymerization of Propylene with *ansa*-Dimethylsilylene(fluorenyl)(cumylamido) Titanium Complexes

**DOI:** 10.3390/polym9040131

**Published:** 2017-04-05

**Authors:** Huajin Wang, Xinwei Wang, Yanjie Sun, Hailong Cheng, Takeshi Shiono, Zhengguo Cai

**Affiliations:** 1 State Key Laboratory for Modification of Chemical Fibers and Polymer Materials, College of Materials Science and Engineering, Donghua University, Shanghai 201620, China; hj.wang518@163.com (H.W.); syj-178@163.com (Y.S.); chl-111@126.com (H.C.); 2 State Key Laboratory of Polyolefins and Catalysis, Shanghai Research Institute of Chemical Industry, Shanghai 200062, China; 13917937459@163.com; 3 Department of Applied Chemistry, Graduate School of Engineering, Hiroshima University, 1-4-1 Kagamiyama, Higashi-Hiroshima 739-8527, Japan

**Keywords:** constrained geometry catalysts, propylene, living polymerization, stereospecificity

## Abstract

A series of *ansa*-silylene(fluorenyl)(amido) titanium complexes (**1a**–**1c**, **2a**, and **2b**) bearing various substituents on the amido and fluorenyl ligands are synthesized and characterized by elemental analysis, ^1^H NMR, and single crystal X-ray analysis. The coordination mode of the fluorenyl ligand to the titanium metal is η^3^ manner in each complex. The propylene polymerization is conducted with these complexes at 0 and 25 °C in a semi batch-type method, respectively. The catalytic activity of **1a**–**1c** bearing cumyl-amido ligand is much higher than that of **2a** and **2b** bearing naphthyl group in amido ligand. High molecular weight polypropylenes are obtained with narrow molecular weight distribution, suggesting a living nature of these catalytic systems at 0 °C. The polymers produced are statistically atactic, regardless of the structure of the complex and the polymerization temperature.

## 1. Introduction

Half-sandwich group 4 metal complexes (usually named constrained geometry catalysts, CGC) having an *ansa*-monocyclopentadienylamido ligand (CpA) have captured significant attention due to their various copolymerization abilities and stereospecificity as olefin polymerization catalysts [1,2,3,4]. The replacement of the Cp ligand by the fluorenyl ligand was effective for the improvement of polymerization activity, copolymerization ability, syndiospecificity, and living polymerization nature to produce novel random and block copolymers with controlled microstructure [5,6,7,8,9,10]. The highly syndiospecific polymerization of α-olefin was also achieved with *C*_s_-symmetric Ti or Zr complexes by the introduction of alkyl groups on the fluorenyl ligand [11,12,13,14,15]. Although *C*_1_-symmetric metallocene catalysts normally give highly isospecific polypropylene (PP) [16,17], *C*_1_-symmetric CGC catalysts exert a less positive influence on the isospecific polymerization of propylene. For example, Fink et al. showed that *C*_1_-symmetric CpA complex by the use of naphthylamido ligand produces isospecific PP with isotactic triad (mm) of 0.63 [18]. Therefore, it is important to investigate strategies for understanding the relationship between the complex structure and the stereospecificity. 

We have realized syndiospecific living homo- and copolymerization of α-olefins with norbornene using *C*_s_-symmetric *ansa*-(fluorenyl)(amido)dimethyltitanium complexes with high activity [19,20,21,22]. We also synthesized *C*_1_-symmetric complex and the other compounds, and found that the change of the structure of amido ligand did not affect livingness of catalytic system; however, the highest mm value was 0.61 [23,24].

**Figure 1:**
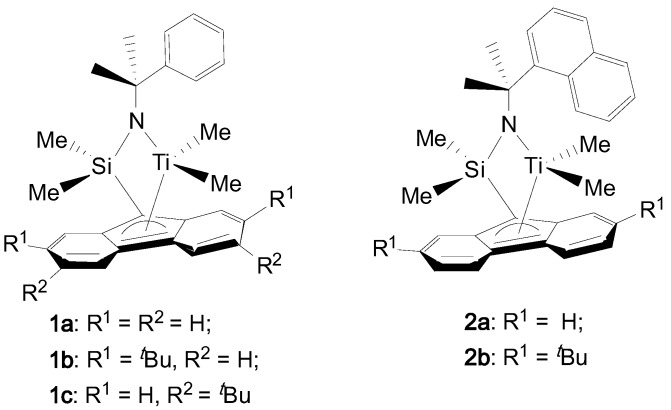
Chemical diagrams of the *ansa*-(fluorenyl)(amido) titanium complexes studied.

In this paper, we synthesized a series of new (fluorenyl)(amido) titanium complexes (**1** and **2** in Figure 1) and investigated the substitute effects of the bulkiness on the amido and fluorenyl ligands in propylene polymerization. 

## 2. Experimental Section

### 2.1. Materials

All operations were performed under nitrogen gas using standard Schlenk techniques, and all solvents were purified by PS-MD-5 (Innovative Technology, Oldham, UK) solvent purification system. Research grade propylene was purified by passing it through a dehydration column of ZHD-20 (Zhonghuida, Dalian, China) and a deoxidation column of ZHD-20A (Zhonghuida, Dalian, China) before used. Modified methylaluminoxane (MMAO) was donated by Tosoh-Finechem Co. (Tokyo, Japan). The ligands and complexes were prepared according to the literature procedure [14,15,24]. We have previously reported the synthesis of complex **1a [24]**. In this work, we report the molecular structure and crystallographic data of complex **1a**.

### 2.2. Synthesis of Complexes

#### 2.2.1. Synthesis of (2,7-di-tBu)fluorenyl-cumylamido Titanium Complex (**1b**)

MeLi (1.6 M in ether, 15 mL, 24 mmol) was added dropwise at 0 °C into a solution of (2,7-di-tBu)fluorenyl-cumylamido ligand (2.82 g, 6 mmol) in 60 mL of diethylether. The resultant orange solution was stirred at r.t. for 4 h, then was added to a solution of TiCl_4_ (0.66 mL, 6 mmol) in 30 mL hexane at room temperature in the stirring condition for 2 h. The solvent was removed, and the residue was extracted with hexane (150 mL). The hexane solution was concentrated to 50 mL and cooled at −30 °C for some days to get **1b** as red crystals (0.92 g, 28%).

^1^H NMR (CDCl_3_): 8.04(d, 2H, Flu), 7.63(s, 2H, Flu), 7.58(d, 2H, Flu), 7.41(d, 2H, Cph), 7.30(t, H, Cph), 7.22(d, 1H, Cph), 1.89(s, 6H, CCH_3_), 1.39(s, 18H, Flu-t-Bu), 0.42(s, 6H, SiCH_3_), –0.39(s, 6H, TiCH_3_). Anal. Calc. for C_34_H_47_NSiTi: C, 74.83; H, 8.68; N, 2.57. Found: C, 74.64; H, 8.85; N, 2.67.

#### 2.2.2. Synthesis of (3,6-di-tBu)fluorenyl-cumylamido Titanium Complex (**1c**)

Complex **1c** was synthesized in a similar way to that for **1b**, and yellow powders were obtained in 28% yield.

^1^H NMR (CDCl_3_): 8.07(s, 2H, Flu), 7.58(d, 2H, Flu), 7.46(d, 2H, Flu), 7.42(d, 2H, Cph), 7.30(t, 2H, Cph), 7.23(d, 1H, Cph), 1.88(s, 6H, CCH_3_), 1.48(s, 18H, Flu-t-Bu), 0.35(s, 6H, SiCH_3_), 0.38(s, 6H, TiCH_3_). Anal. Calc. for C_34_H_47_NSiTi: C, 74.83; H, 8.68; N, 2.57. Found: C, 74.60; H, 8.84; N, 2.65.

#### 2.2.3. Synthesis of Fluorenyl-(1-methyl-(1-naphthyl)ethyl)amido Titanium Complex (**2a**)

Complex **2a** was synthesized in a similar way to that for **1b**, and red crystals were obtained in 12% yield.

^1^H NMR (CDCl_3_): 8.58(d, 1H, Naph), 8.06(d, 2H, Flu), 7.87(d, 1H, Naph), 7.79(d, 1H, Naph), 7.63(d, 2H, Flu), 7.29–7.56(m, 4H, Naph; 4H, Flu), 2.15(s, 6H, CCH_3_), 0.03(s, 6H, SiCH_3_), −0.19(s, 6H, TiCH_3_). Anal. Calc. for C_30_H_33_NSiTi: C, 74.52; H, 6.88; N, 2.90. Found: C, 74.77; H, 7.02; N, 2.31.

#### 2.2.4. Synthesis of (2,7-di-tBu)fluorenyl-(1-methyl-(1-naphthyl)ethyl)amido Titanium Complex (**2b**)

Complex **2b** was synthesized in a similar way to that for **1b**, and red crystals were obtained in 11% yield.

^1^H NMR (CDCl_3_): 8.70(d, 1H, Naph), 8.04(d, 2H, Flu), 7.86(d, 1H, Naph), 7.78(d, 1H, Naph), 7.58(t, 2H, Flu; 3H, Naph), 7.44(m, 2H, Flu), 7.30(t, 1H, Naph), 2.13(s, 6H, CCH_3_), 1.39(s, 18H, Flu-t-Bu), 0.09(s, 6H, SiCH_3_), −0.23(s, 6H, TiCH_3_). Anal. Calc. for C_38_H_49_NSiTi: C, 76.61; H, 8.29; N, 2.35. Found: C, 76.39; H, 8.38; N, 2.19.

### 2.3. Polymerization Procedure

Polymerization was performed in a 100 mL glass reactor equipped with a magnetic stirrer and carried out using semi-batch method. The reactor was charged with prescribed amounts of MMAO/2,6-di-*tert*-butyl-4-methyl phenol (BHT) and solvent (30 mL). After the solution of cocatalyst was saturated with gaseous propylene under atmospheric pressure, polymerization was started by the addition of 1 mL solution of the Ti complex (20 μmol) in reactor, and the consumption rate of propylene was monitored by a mass flow meter. Polymerization was conducted for a certain time and terminated with acidic methanol. The polymers obtained were adequately washed with methanol and dried under vacuum condition at 80 °C for 6 h to be a constant weight.

### 2.4. Analytical Procedure

Single crystal of complex was obtained from a solution of hexane at −30 °C. The single crystals were mounted under nitrogen atmosphere at low temperature, and data collection was made on a Bruker APEX2 diffractometer using graphite monochromated with Mo K*α* radiation (*λ* = 0.71073 Å). The SMART program package was used to determine the unit cell parameters. The absorption correction was applied using SADABS program [25]. All structures were solved by direct methods and refined on *F*^2^ by full-matrix least-squares techniques with anisotropic thermal parameters for non-hydrogen atoms. Hydrogen atoms were placed at calculated positions and were included in the structure calculation. Calculations were carried out using the SHELXS-97, SHELXL-2014, or Olex2 program [26,27,28,29,30,31,32]. Crystallographic data are summarized in Table 1, and CIF files and check are provided in the Supporting Information. 

Molecular weights and molecular weight distributions of polymers were measured by a polymer laboratory PL GPC-220 chromatograph (Agilen, Santa Clara, CA, USA) equipped with one PL1110-1120 column and two PL MIXED-B 7.5 mm × 300 mm columns at 150 °C using 1,2,4-trichlorobenzene as solvent and calibrated by polystyrene standards. The ^1^H NMR spectra of complexes and ^13^C NMR spectra of PPs were measured on a Bruker Asend™ 600 spectrometer (Bruker, Karlsruhe, Germany). Differential scanning calorimeter (DSC) analyses were performed on TA Q2000 instrument (Waters, New Castle, DE, USA), and the DSC curves of the samples were recorded under a nitrogen atmosphere at a heating rate of 10 °C/min from 40 to 200 °C.

## 3. Results and Discussion 

### 3.1. Molecular Structure of Complexes

**Figure 2:**
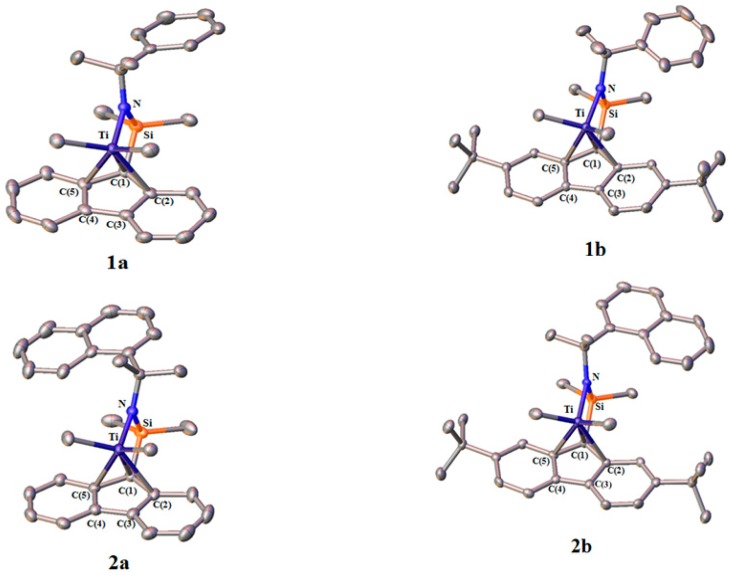
Structure of fluorenylamidotitanium complexes **1a**, **1b**, **2a**, and **2b**. Hydrogen atoms are omitted for clarity. Atoms are drawn at the 40% probability level.

The complexes were synthesized in good yield using one-pot reaction of the corresponding ligand with a two-fold excess of MeLi and TiCl_4_ in hexane. The molecular structure of complexes determined by single crystal X-ray analysis are shown in Figure 2, and the selected bond lengths and angles of complexes are shown in Table 2. The bond lengths between the titanium and the fluorenyl carbons (C(1), C(2), C(3), C(4), and C(5)) are almost the same as that of previously reported *C*_s_-symmetric (fluorenyl)(amido) titanium complexes [24], which suggests that the fluorenyl ligand is coordinating to the titanium metal with η^3^-form, irrespective of the substituent structure.

In each complex, no symmetry plane or axis was observed, indicating the *C*_1_-symmetric nature in solid state. On the other hand, the ^1^H NMR spectra of all the complexes showed that the methyl groups bonded to the Si and Ti atoms are equivalent, which can be explained by the fast rotation of the admido ligand on the NMR time-scale at room temperature. 

### 3.2. Propylene Polymerization

Propylene polymerizations were conducted with **1a**–**1c**, **2a**, and **2b** activated by MMAO in a semi batch-type operation under an atmospheric pressure of propylene in toluene at 0 and 25 °C (Table 3). Cumyl-amido complexes **1a**–**1c** showed much higher activity (1900–2500 kg PP/(mol Ti·h)) than naphthyl-amido complexes **2a** and **2b** (20–120 kg PP/(mol Ti·h)), of which value increased according to the increase of polymerization temperature and was independent of the introduction of *t*-butyl group on the fluorenyl ligand. Significantly lower activity in **2a** and **2b** can be attributed to the steric hindrance of the amido group, where the steric hindrance of naphthyl group (102°/103°) is bigger than the cumyl group (86°/90°) as estimated by Tolman cone angle (Table 4) [33].

**1a**–**1c** gave high molecular weight PP (*M*_n_ > 230,000) for 5 min at 0 °C with narrow molecular weight distribution, and the number of polymer chains (*N*) were about 65–70% of the titanium complex used. These results suggest that the propylene polymerization proceeded in a living manner. The molecular weights of PPs were decreased by raising the polymerization temperature, with broader molecular weight distribution accompanied by the increase of *N* value, indicating that the chain transfer reaction frequently occurred by the improvement of polymerization temperature. The molecular weights of PPs obtained by **2a** and **2b** were lower, but the molecular weight distributions were extremely narrow. The *N* values were about 25% of the titanium used, which indicates poor catalytic efficiency by the introduction of the bulky naphthyl group.

All the PPs obtained were amorphous measured by the DSC analysis. To investigate the substituent effect of the amido group on the stereospecificity, we conducted ^13^C NMR analysis of PPs obtained. The steric pentad distributions are summarized in Table 5. All the polymers obtained in each catalyst system were statistically atactic. The result indicates that the introduction of sterically demanded *N*-alkyl and *t*-butyl groups on the amido and the fluorenyl ligand, respectively, had little influence on the stereospecificity.

## 4. Conclusions

(Fluorenyl)(amido) titanium complexes **1a**–**1c**, **2a**, and **2b** were synthesized and characterized by ^1^H NMR, elemental analysis, and single crystal X-ray analysis. All synthetic catalysts are *C*_s_-symmetric in solution, irrespective of the bulkiness of the amido ligand. The cumyl-amido complexes **1a**–**1c** showed much higher activity than the naphthyl-amido complexes **2a** and **2b** because of the steric hindrance of the amido group. The modification of the structure did not change the livingness of the catalytic system, whereas the change of ligand structure was not efficient for the isospecificity of CGC catalyst.

## Figures and Tables

**Table 1:** Crystallographic data and parameters for **1a**, **1b**, **2a**, and **2b**.

Complex	1a	1b	2a	2b
deposition numbers	CCDC 1539490	CCDC 1539499	CCDC 1540563	CCDC 1539502
formula	C_26_H_31_NSiTi	C_34_H_47_NSiTi	C_30_H_33_NSiTi	C_38_H_49_NSiTi
formula weight	433.51	545.71	483.56	595.77
crystal system	Monoclinic	Triclinic	Monoclinic	Triclinic
space group	*P*1 2_1_/*c*1	*P*$\overset{-}{1}$	*P*1 2_1_/*c*1	*P*$\overset{-}{1}$
*a* (Å)	8.8160(15)	10.0609(13)	12.742(3)	10.3445(13)
*b* (Å)	27.430(5)	11.5953(15)	17.562(4)	11.8424(16)
*c* (Å)	9.9361(18)	15.4910(3)	11.404(2)	15.4920(3)
*β*(deg)	108.372(4)	100.124(3)	95.269(4)	99.822(4)
*V*(Å^3^)	2280.3(7)	1561.1(4)	2541.1(9)	1636.8(4)
*Z*	4	2	4	2
*F*(000)	920	588	1024	640
*D*_calcd_. (g·cm^−3^)	1263	1161	1264	1209
μ (mm^−^^1^)	0.440	0.334	0.402	0.325
theta range for data collection	2.284° to 30.611°	2.002° to 30.542°	1.980° to 30.772°	1.965° to 25.499°
reflections collected	23,087	15,856	25,451	11,599
independent reflections	7002	9413	7920	6105
final R indices [*I* > *2δ*(*I*)]	[R(int) = 0.1147]	[R(int) = 0.0235]	[R(int) = 0.0610]	[R(int) = 0.0569]
	R1 = 0.0512, wR2 = 0.0963	R1 = 0.0398, wR2 = 0.0940	R1 = 0.0455, wR2 = 0.1040	R1 = 0.0508, wR2 = 0.1051

**Table 2:** Selected bond lengths (Å) and bond angles (°) for related complexes.

Parameter	1a	1b	2a	2b
Ti(1)–C(1)	2.241(2)	2.250(14)	2.260(19)	2.267(3)
Ti(1)–C(2)	2.409(3)	2.384(14)	2.414(19)	2.413(3)
Ti(1)–C(3)	2.586(3)	2.599(14)	2.556	2.576(3)
Ti(1)–C(4)	2.594(3)	2.615(15)	2.538	2.569(3)
Ti(1)–C(5)	2.398(2)	2.430(15)	2.396(19)	2.394(3)
Ti(1)–N(1)	1.919(2)	1.922(12)	1.922(17)	1.920(2)
Ti(1)–Si(1)	2.836(9)	2.828(6)	2.850	2.848(11)
N(1)–Ti(1)–C(1)	78.35(9)	78.56(5)	78.01(7)	77.74(11)
Ti(1)–N(1)–Si(1)	100.88(10)	100.40(6)	101.30(8)	101.76(12)
N(1)–Si(1)–C(1)	93.66(10)	94.20(6)	93.75(8)	93.88(13)

**Table 3:** Propylene polymerization with Ti complexes **1a**–**2b** activated by MMAO ^a^.

Entry	Cat.	Temp. (°C)	Time (min)	Yield (g)	Activity ^b^	*M*_n_^c^ (×10^4^)	*M*_w_/*M*_n_^c^	*N* ^d^ (μmol)
1	**1a**	0	5	3.21	1930	25.2	1.49	13
2	**1b**	0	5	3.46	2080	23.9	1.37	14
3	**1c**	0	5	3.59	2150	26.1	1.58	14
4	**1a**	25	5	4.05	2430	16.8	2.05	24
5	**1b**	25	5	4.06	2430	19.4	1.70	21
6	**1c**	25	5	4.21	2530	16.6	1.93	25
7	**2a**	0	12	0.271	69	5.18	1.18	5.2
8	**2b**	0	12	0.084	21	1.73	1.16	4.9
9	**2a**	25	12	0.474	119	4.81	1.43	9.9
10	**2b**	25	12	0.362	91	4.38	1.27	8.3

^a^ Polymerization conditions: toluene = 30 mL, Ti = 20 μmol, Al/Ti = 600, propylene = 1 atm; ^b^ Activity in kg of PP/(mol of Ti·h); ^c^ Number-average molecular weight and molecular weight distribution determined by gel-permeation chromatography (GPC) using universal calibration; ^d^ Calculated from yield and *M*_n_.

**Table 4:** Tolman’s cone angle of amido groups of complexes **1a**, **1b**, **2a**, and **2b**.

Catalyst	Fluorenyl	*N*-alkyl	θ (°)
**1a**	Flu	Ph	86
**1b**	2,7-di-*t*Bu-Flu	Ph	90
**2a**	Flu	Naphthyl	103
**2b**	2,7-di-*t*Bu-Flu	Naphthyl	102

**Table 5:** Steric pentad distributions for samples in Table 3 (entries 4–6 and 10).

Entry	Stereosequence distribution ^a^								
	mmmm	mmmr	rmmr	mmrr	mmrm + rmrr	rmrm	rrrr	mrrr	mrrm
4	0.04	0.05	0.06	0.22	0.22	0.13	0.09	0.14	0.05
5	0.02	0.03	0.04	0.18	0.22	0.11	0.15	0.19	0.06
6	0.03	0.05	0.03	0.13	0.20	0.14	0.16	0.21	0.05
10	0.12	0.10	0.08	0.29	0.16	0.05	0.03	0.08	0.09

^a^ Determined by ^13^C NMR spectroscopy.

## Supplementary Materials

The following are available online at www.mdpi.com/2073-4360/9/4/131/s1.

## References

[1] Stevent J.C., Timmers F.J., Wilson D.R., Schmidt G.F., Nickias P.N., Rosen R.K., Knight G.W., Lai S.Y. (1991). Constrain Geometry Addition Polymerization Catalysts, Processer for Their Preparation, Precursors Therefor, Methods of use, and Novel Polymers Formed Therewith.

[2] Canich J.A.M. (1993). Monocyclopentadienyl Titanium Metal Compounds for Ethylene-α-Olefin-Copolymer Production Catalysts. U.S. Patent.

[3] Mcknight A.L., Waymouth R.M. (1998). Group 4 *ansa*-cyclopentadienyl-amido catalysts for olefin polymerization. Chem. Rev..

[4] Braunschweig H., Breitling F.M. (2006). Constrained geometry complexes-synthesis and applications. Coord. Chem. Rev..

[5] Nakayama Y., Sogo Y., Cai Z. (2013). Copolymrization of ethylene with 1,1-disubstituted olefins catalyzed by *ansa*-(fluorenyl)(cyclododecylamido) dimethyltitanium complexes. J. Polym. Sci. Part A.

[6] Xu G. (1998). Copolymerization of ethylene with styrene catalyzed by the [η^1^:η^5^-*tert*-butyl(dimethylfluorenylsilyl)amido] metthyltitanium “Cation”. Macromolecules.

[7] Kirillov E., Razavi A., Carpentier J. (2006). Syndiotactic-enriched propylene-styrene copolymer using fluorenyl-based half-titanocene catalysts. J. Mol. Catal. Part A.

[8] Shiono T., Sugimoto M., Hasan T. (2008). Random copolymerization of norbornene with higher 1-alkene with *ansa*-fluorenylamidodimethyltitanium catalyst. Macromolecules.

[9] Shiono T., Suginoto M., Hasan T., Cai Z. (2013). Facile synthesis of hydroxy-functionalized cycloolefin copolymer using ω-alkenylaluminium as a comonomer. Macromol. Chem. Phys..

[10] Tanaka R., Ikeda T., Nakayama Y., Shiono T. (2015). Pseudo-living copolymerization of norbornene and ω-alkynelborane—Synthesis of monodisperse functionalized cycloolefin copolymer. Polymer.

[11] Razavi A., Thewalt U. (2001). Preparation and crystal structures of the complexes (η^5^-C_5_H_3_TMS-CMe_2_-η^5^-C_13_H_8_)MCl_2_(M = Hf, Zr or Ti): Mechanistic aspects of the catalytic formation of a isotactic-syndiotactic stereoblock-type polypropylene. J. Organomet. Chem..

[12] Busico V., Cipullo R., Cutillo F., Talarico G., Razavi A. (2003). Syndiotactic poly(propylene) from [Me2Si(3,6-di-*tert*-butyl-9-fluorenyl) (*N*-*tert*-butyl)]TiCl_2_-based catalysts: Chain-end or enantiotopic-sites stereocontrol?. Macromol. Chem. Phys..

[13] Irwin L.J., Miller S.A. (2005). Unprecedented syndioselectivity and syndiotactic polyolefin melting temperature: Polypropylene and poly(4-methyl-1-pentene) from a highly active, sterically expanded η^1^-fluorenyl-η^1^-amido zirconium complex. J. Am. Chem. Soc..

[14] Nishii K., Hagihara H., Ikeda T., Akita M., Shiono T. (2006). Stereospecific polymerization of propylene with group 4 *ansa*-fluorenylamidodimethyl complexes. J. Organomet. Chem..

[15] Miller S.A., Bercaw J.E. (2004). Highly Stereoregular syndiotactic polypropylen formation with metallocene catalysts via influence of distal ligand substituents. Organometallics.

[16] Bader M., Marquet N., Kirillov E., Roisnel T., Razavi A., Lhost O., Carpentier J.F. (2012). Old and new *C*_1_-symmetric group 4 metallocenes {(R_1_R_2_C)-(R^2^’R^3^’R^6^’R^7^-Flu)(3-R^3^-5-R^4^-C_5_H_2_)}ZrCl_2_: From highly isotactic polypropylenes to vinyl end capped isotactic-enriched oligomers. Organometallics.

[17] Esteb J.J., Chien J.C.C.W., Rausch M.D. (2003). Novel *C*_1_ symmetric zirconocenes containing substituted indenyl moieties for the stereoregular polymerization of propylene. J. Organomet. Chem..

[18] Kleinschmidt R., Griebenow Y., Fink G. (2000). Stereospecific propylene polymerization using half-sandwich metallocene/MAO systems: A mechanistic insight. J. Mol. Catal. A Chem..

[19] Cai Z., Nakayama Y., Shiono T. (2006). Living random copolymerization of propylene and norbornene with *ansa*-fluorenylamidodimethyltitanium complex: Synthesis of novel syndiotactic polypropylene-*b*-poly(propylene-ran-norbornene). Macromolecules.

[20] Cai Z., Harada R., Nakayama Y., Shiono T. (2010). Highly active living random copolymerization of norbornene and 1-alkene with *ansa*-fluorenylamidodimethyltitanium derivative: Substituent effects on fluorenyl ligand. Macromolecules.

[21] Cai Z., Ikeda T., Akita M., Shiono T. (2005). Substituent effects of *tert*-butyl groups on flurenyl ligand in syndiospecific living polymerization of propylene with *ansa*-fluorenylamidodimethyltitanium complex. Macromolecules.

[22] Cai Z., Ohmagari M., Nakayama Y., Shiono T. (2009). Highly active syndiospecific living polymerization of higher 1-alkene with *ansa*-fluorenylamidodimethyltitanium complex. Macromol. Rapid Commun..

[23] Cai Z., Su H., Nakayama Y., Shiono T., Akita M. (2014). Synthesis of *C*_1_ symmetrical *ansa*-cyclopentadienylamidotitanium complexes and their application for living polymerization of propylene. J. Organomet. Chem..

[24] Tanaka R., Yanase C., Cai Z. (2016). Structure-stereospecificity relationships of propylene polymerization using substituted *ansa*-silylene(fluorenyl)(amido) titanium complexes. J. Organomet. Chem..

[25] Sheldrick G.M. (1996). SADABS: An Empirical Absorption Correction Program for Area Detector Data.

[26] Sheldrick G.M. (2008). Crystal structure refinement with SHELXL. Acta Crystallogr..

[27] Sheldrick G.M. (2015). A short history of SHELX. Acta Cryst..

[28] Dolomanov O.V., Bourhis L.J., Gildea R.J., Howard J.A.K., Puschmann H. (2009). OLEX2: A complete structure solution, refinement and analysis program. J. Appl. Cryst..

[29] (2002). SAINT+, Vdrsion 6.22a.

[30] (2009). SAINT+, Vdrsion v7.68A.

[31] (2002). SHELXTL NT/2000, Version 6.1.

[32] Sheldrick G.M. (2015). Crystal structure refinement with SHELXL. Acta Crystallogr. Sect. C.

[33] Tolman C.A., Seidel W.C., Gosser L.W. (1974). Formation of three-coordinate Nike(0) complexes by phosphorus ligand dissociation from NiL_4_. J. Am. Chem. Soc..

